# Sorption-Related Characteristics of Surface Charred Spruce Wood

**DOI:** 10.3390/ma11112083

**Published:** 2018-10-24

**Authors:** Maija Kymäläinen, Hannu Turunen, Petr Čermák, Saara Hautamäki, Lauri Rautkari

**Affiliations:** 1 Department of Bioproducts and Biosystems, School of Chemical Engineering, Aalto University, P.O. Box 16300, FI-00076 Aalto, Finland; saara.hautamaki@aalto.fi (S.H.); lauri.rautkari@aalto.fi (L.R.); 2 South-Eastern Finland University of Applied Sciences—XAMK, Mikpolis-Research Unit, P.O Box 68, FI-50101 Mikkeli, Finland; hannu.turunen@xamk.fi; 3 Department of Wood Science, Faculty of Forestry and Wood Technology, Mendel University in Brno, Zemědělská 3, 613 00 Brno, Czech Republic; petr.cermak.und@mendelu.cz

**Keywords:** char, surface modification, thermal modification, wood, wood char

## Abstract

Surface charring of wood is a one-sided thermal modification process that can be used to create a hydrophobic, durable surface to exterior claddings. Spruce (*Picea abies* L.) wood samples were charred with a hot plate and several time-temperature combinations while using simultaneous surface compression. Temperature profile, water sorption, cupping after water exposure and density profile were measured. Furthermore, changes in the microstructure and surface functional groups were investigated by scanning electron microscopy and photoacoustic FT-IR spectroscopy. Results show that surface charring notably improves the hydrophobicity measured by contact angle, water floating and dynamic vapour sorption. Increased holding time during charring reduced the sorption but at the same time increased the dimensional instability measured by cupping. The density profile showed a shifting density peak with more severe modification regimes, indicating a more porous surface. The PAS-FTIR showed increased aromaticity of the surface that was also present in the pyrolysis zone beneath the surface in samples modified with longer holding time. Higher modification temperature affected the sorption as well as cupping positively but it is possible similar results can be obtained with lower temperature and longer holding time.

## 1. Introduction

Growing interest in green construction increases the need for sustainable renewable materials. Wood is an ecological, low-cost choice for outside claddings of buildings. With proper care and maintenance, wooden façades are also a long-lasting, low-impact option that are easy to recycle and dispose of in the end of their service life. However, as the energy consumption of the building use phase decreases due to more energy efficient construction, the relevance of material choices increases [1,2,3]. The building life may span between 20 to 100 years [4] with recurring façade maintenance operations such as recoating forming a major share of the life cycle costs. Wood is susceptible to weathering caused by UV-radiation, temperature and moisture changes, as well as rain, snow and ice. Weathering causes erosion of the wood surface and may lead to further damage caused by biological factors such as fungal activity. To protect the wood from weathering, it should be coated, which adds to the environmental burden and overall costs of the cladding. Renewal is required every 2–15 years, depending mainly on the opacity of the finish [5,6]. The location of the wall also influences durability, as walls facing south (on the northern hemisphere) will degrade faster, as will façades in humid climates compared to drier climates [6]. 

Wood modification chemically or thermally changes the structure of wood into a more durable form. Modified wood is characterized by decreased moisture sorption and, consequently, increased dimensional stability and resistance to biological degradation [7,8]. Thermal processes utilize temperatures below 300 °C [3] and limit oxidation reactions. Although thermally modified (TM) wood is environmentally friendly and suitable for many applications, there are some downsides. Regardless of the process used, TM wood exhibits low physical strength that prevents its use in load-bearing structures [3,9,10]. The colour will fade to grey in outdoors exposure, cracking and cupping takes place and maintenance interval (recoating) cannot be significantly increased compared to unmodified wood [11,12,13,14]. The modification methods also tend to be quite time-consuming [15,16] adding to the cost of the product. As an option, wood could be modified only from the exposed surface, sparing time and costs and preserving the structural properties of wood. One-sided surface charring has been utilized traditionally in Japan [17]. In this technique, the wood is burned with naked flame or a heated iron pad and the result is said to be practically maintenance-free and last for decades.

Thermal degradation of wood is a complex set of overlapping reactions. Generally, an endotherm characterizing the evaporation of water can be located between 110 and 130 °C. This is followed by a set of exotherms at around 180 °C, where destruction of hydrogen bonds takes place and 220–335 °C with cleavage of glucosidic linkages and short-chained lignins, as well as decomposition of hemicelluloses and cellulose [18,19]. Most of the hemicelluloses have decomposed at approximately 300 °C and the most rapid rate of decomposition owing to cellulose and lignin takes place at approximately 360 °C, while the remaining lignin continues to decompose above 400 °C [20]. The char front moves into the wood in layers. The evaporation zone is followed by a region where pyrolysis takes place between 130 to about 288 °C. The structure of this pyrolysis zone is likely to be very similar to TM wood. Hemicelluloses react first and liberate acetic acid that further catalyse depolymerisation of both hemicelluloses and amorphous cellulose [21,22]. The decomposition leads to reduced hygroscopicity because of destruction of hydroxyl sites. There is also slight increase in aromatization of lignin in these temperatures that increases cross-linking, as well as decreased expansion potential of cellulose microfibrils [21] that, along with increased crystallinity due to preferential degradation of amorphous network, increases dimensional stability [22]. Actual wood charring begins at 288–300 °C [23,24,25], coinciding with the exotherm at 270–325 °C. In air, the reactions are initiated at lower temperatures but take place mainly in the same temperature regions regardless [23,24,26,27]. The char layer remaining on the wood surface will be hydrophobic, cross-linked and aromatic but also porous and brittle, at least in the lower part of the temperature range [28,29]. Capillary absorption can take place and a cracked surface may lead to water penetrating into the unmodified inner wood [30].

Spruce is a commonly used material for exterior claddings in Finland. In order to investigate the effect of one-sided charring on surface properties of spruce in relation to service life and resistance to weathering, several characteristics were studied. The results form a part of a project aiming to develop a sustainable cladding material with improved performance and reduced need for maintenance compared to other available wooden materials by utilizing surface charring.

## 2. Materials and Methods

### 2.1. Preparation of Samples

For the experiments, we used Finnish spruce wood (*Picea abies* (L.) Karst.), which is the most common wood species for outside claddings in Finland. The samples were sawn from sapwood boards conditioned at 65% RH and 20 °C, giving them a moisture content (MC) of about 12%. The boards were planed once from either of the tangential sides using a surface planer and cut to 50 × 100 mm pieces by a band/circular saw. The thickness of the samples varied between 20 and 25 mm. Small knots with the maximum diameter of 2 mm were allowed. The orientation of the annual rings was horizontal to test surface and kept as even as possible and the samples were taken from boards with similar densities to minimize variation.

The samples were charred using a made-to-order heated steel plate (Meyer-vastus, Finland) and a laboratory press (Weverk AB, Karlstad, Sweden), as the aim was also to investigate the effect of simultaneous surface densification. For this, the press was set to a surface pressure of 1 MPa. The time-temperature regimes are presented in Table 1. The plate was pre-heated to a desired peak temperature prior to contact with the sample, after which the press was closed for the set holding time. Ignition was avoided, thus affecting the choice of durations at a certain temperature. All the samples were charred on heartwood side (pith side) of the boards and stored at 65% RH, 20 °C before further experimental use. 

### 2.2. Wettability Measurements

The surface contact angle of the laboratory samples was measured with sessile drop method (CAM2000, KSV Instruments Ltd., Helsinki, Finland), using a drop size of 8.7 µL and a time of 60 s, with one frame per second recorded. Ten samples were measured per treatment, with three randomly selected points per each sample.

The surface wettability was measured also by water floating according to EN 927-5 [31]. The sample sides were sealed with a double layer of alcoxy silicone to avoid wetting of any other surface than the modified one. The mass of the samples (10 per treatment) was measured before placing them into a container with water at room temperature. The weighing was repeated at 6, 12, 24, 36, 48 and 72 h, after which the experiment was terminated. The cupping of the samples was measured at the time of the weighing before and during the experiment. The cup was measured once from the back (bark side) and the modified surface (pith side) at the length-wise centre using a digital dial indicator (Mitutoyo IDU-25 Digimatic indicator, Mitutoyo Corp., Kawasaki, Japan) to an accuracy of 0.02 mm. The statistical significance of observed changes was analysed with IBM SPSS Statistics 25, using paired samples *t*-test, one way ANOVA and generalised linear model.

### 2.3. DVS Sorption Measurements

To examine the moisture adsorption properties of charred wood, thin flakes were extracted from the surface and the pyrolysis layers of the samples with a razor blade and ground to a coarse powder (Micro Hammer Mill, Culatti AG, Steinen, Switzerland). Pyrolysis zone material was carefully avoided in the surface samples and unmodified wood in the pyrolysis zone samples. The measurements were made with a dynamic vapour sorption (DVS) apparatus (DVS Intrinsic, Surface Measurement Systems, London, UK). The device produces very accurate sorption isotherms, utilizing a microbalance in a carefully controlled atmosphere of nitrogen and water vapour. About 20 mg of the powder was measured on the balance. The method used consisted of an initial drying step (to 0% EMC) followed by 12 h flushing at 95% RH. The actual measurement cycle was 0-95-0% RH in 5% steps. The next step was reached once the change in sample mass was less than 0.002% for a period of 10 min. The pre-flushing was implemented to simulate the effect of an additional cycle and remove possible volatile degradation products that may have an effect on sample mass and, therefore, the isotherms [8]. It took from two to three days to complete the cycle, depending on the sample. One cycle was measured per sample.

### 2.4. FT-IR Analysis

Small flakes, cut as thin as possible with a razor blade, were extracted from the surface and the pyrolysis zones of the samples for the photoacoustic Fourier infrared (PAS-FTIR) analysis (Bio-Rad FTS 6000 Spectrometer, Cambridge, MA, USA). A background spectrum of carbon black was run before analysing the samples. A wavelength range of 4000–480 cm^−1^ was used and each measurement consisted of a minimum of 350 scans. The data was processed with Win-IR Pro 3.4 software (Digilab, Randolph, MA, USA). The spectral data was averaged between two or three repeats and normalized to peak at 1508 cm^−1^ as in Faix & Böttcher [32]. 

### 2.5. Microstructural Analysis

The reference, along with surface compressed and uncompressed samples 300-30 and 400-3 were chosen to represent unmodified, moderate and severe modification regimes. The samples were prepared with a sliding microtome (Nahita ZFP011, Auxilab S.L., Navarra, Spain) to a thickness of 45–60 µm. To ease the cutting, samples were first soaked in distilled water for complete wetting and during cutting wetted with a water-glycerol-alcohol mixture. The samples were mounted on pin stubs with carbon tape and sputter coated (Emitech K100X, EM Technologies Ltd., Ashford, UK) with palladium/gold for 1:30 min (30 mA current) prior to imaging with scanning electron microscope (Zeiss Sigma VP FE-SEM (Carl Zeiss Microscopy GmbH, Jena, Germany).

### 2.6. Density Profile

The reference along with samples 250-60, 300-30 and 350-5 were subjected to a density profile analysis. The samples (conditioned at 20 °C, 65% RH) were measured with X-ray Density Profile Analyzer—DPX300 with a measuring step of 0.05 mm. The samples were measured four times.

## 3. Results

### 3.1. Temperature Profile

**Figure 1:**
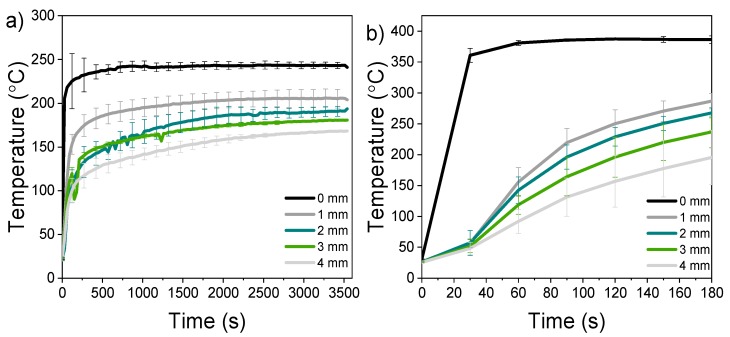
Temperature profiles measured from (**a**) samples modified at 250 °C, 60 min and (**b**) 400 °C, 3 min, average of three. See Table 1 for sample codes.

The temperature profile measured during the charring revealed that the effect of heat applied to the surface extended only parts of a millimetre into the wood (Figure 1). A temperature of 130 °C was set as the limit for the beginning of thermal degradation reactions. With the sample 250-60 (modified at 250 °C for a duration of 60 min) this temperature was measured at a distance of 1 mm after 75 s of exposure. When the exposure temperature exceeded 300 °C, the temperature of 130 °C was reached within about 45 s regardless of set peak temperature (at 1 mm from surface). The temperature of 130 °C was reached in every sample down to the distance of 4 mm from surface in 85–540 s (samples 400-3–250-60, respectively). A temperature of 200 °C was set as the threshold for the formation of a thermally modified layer, corresponding to an average temperature used in thermal modification utilizing steam as a shielding gas (here replaced by moisture evaporating from inside the sample). The sample modified at 250-60 reached 200 °C only at 1 mm from the surface (after 1500 s), whereas the sample 300-10 reached it down to 3 mm from surface (after 200 s). The sample modified at 400-3 did not reach 200 °C at 4 mm within 180 s. The temperature measurements also revealed that the surface temperature was consistently 10–20 °C less than the set point, most likely caused by evaporation of water. The applied pressure crushed the first millimetres of the wood into a single layer, densifying the surface. In the actual compressed samples the temperature increase within the wood was therefore likely to be slightly smaller than in the uncompressed samples.

### 3.2. Microstructural Changes

**Figure 2:**
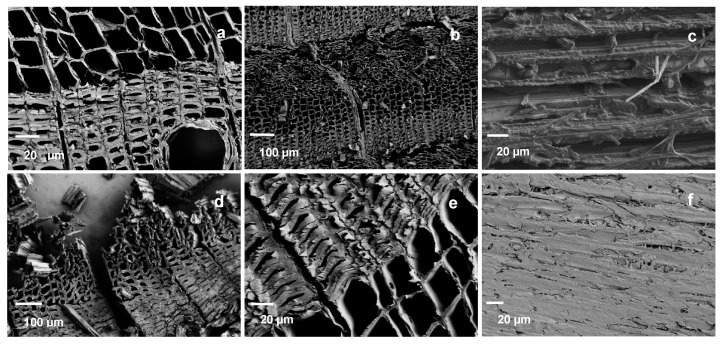
SEM images of (**a**) unmodified spruce, (**b**) compressed transverse pyrolysis zone of 300-30, (**c**) uncompressed tangential charred surface of 300-30, (**d**) compressed transverse charred surface of 400-3, (**e**) uncompressed pyrolysis zone of 400-3, (**f**) compressed tangential charred surface of 400-3.

The pressure applied to the laboratory samples naturally affected the surface properties, therefore also uncompressed samples were imaged. In compressed samples, the top layers of wood cells were crushed, preferentially in the earlywood section while the latewood sections held their form better. Looking at the samples in Figure 2, it can however be seen that the charring had little effect on the cellular structure. The earlywood sections are distorted to a depth of about three annual rings with intact latewood sections in between. There are little or no visible cracks on the cell walls. The tangential surface exposed to heat shows the effect of densification clearly, with the structure and the individual fibres flattened and plasticized (2f vs. 2c).

### 3.3. Surface Wettability 

**Figure 3:**
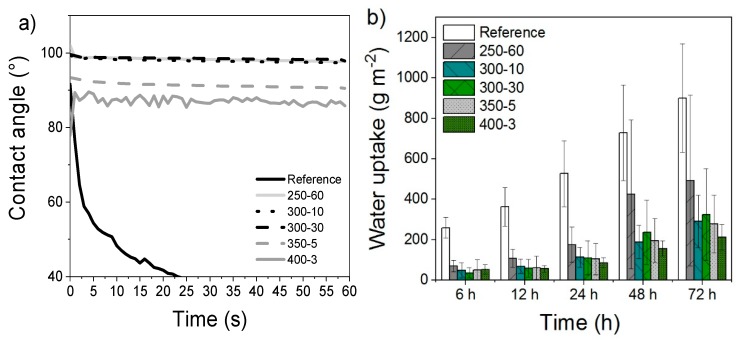
(**a**) Wettability measured by contact angle during a measurement period of 60 s and (**b**) absorption (g m^−2^) of samples measured by 0–72 h water floating. The vertical scale on 3a has been shortened and the standard deviation omitted to better highlight individual differences between modified samples. The figure including standard deviations is presented in Supplementary Figure S1.

The contact angles measured with sessile drop method revealed large differences between reference and modified samples (Figure 3). During the first recorded second, the reference sample contact angle dropped from 92 to 76° while the contact angles of the modified samples were significantly higher and more stable during the measurement period. The samples modified at 250 to 300 °C behaved very similarly with each other, while the contact angles of samples treated at 350 and 400 °C decreased slightly in comparison. Despite the decline, all modified samples performed much better than reference and the applied droplet did not noticeably absorb to the surface during the 60 s measurement period, except with the samples modified at the most severe conditions at 400 °C. The 400-3 samples also showed the highest variation within the 30 measurements. All samples differed significantly (*p* = 0.000) from reference in paired samples *t*-test. 

### 3.4. Absorption by Water Floating 

The water floating experiment similarly revealed important differences between treatments. Figure 3b presents the results as g m^−2^. The laboratory samples modified at 250-60 showed only slight decrease compared to absorption of reference samples. On the other hand, samples modified above 300 °C showed much improved hydrophobicity. At 72 h, the compressed laboratory samples modified at 300–400 °C exhibit only minor differences, though the standard deviations within the 300-30 samples increase more than those of the rest. The paired samples *t*-test showed all but 250-60 differing significantly (*p* = 0.000) from reference. The sample 250-60 also shows very high standard deviations with longer experiment duration.

### 3.5. Sorption Measured by DVS

**Figure 4:**
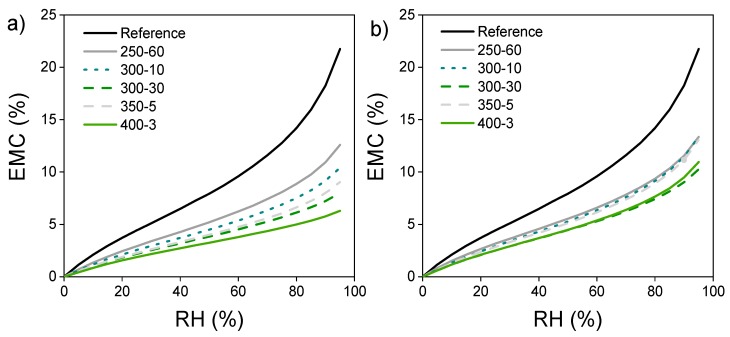
(**a**) Adsorption isotherms of surface and (**b**) pyrolysis zone between 0 and 95% RH.

In the DVS analysis, the adsorption of water vapour decreases with increasing modification temperature (Figure 4a). Sample 350-5 follows the curve of 300-30 but shows increased adsorption at high RH. The pyrolysis zone adsorption curves (Figure 4b) also decrease from reference, indicating changes in the availability of OH-groups. However, the EMC at a certain RH is more constant between different treatments in comparison to the surface samples. The effect of extended holding time is also more evident, the 300-30 pyrolysis zone sample showing similar EMC as 400-3, being the lowest of the treatments at highest RH. Sample 350-5 on the other hand shows higher EMC, closely following the curve of 250-60 that is nearly identical in both the surface and the pyrolysis zones. 

### 3.6. Changes in the Surface Functional Groups

**Figure 5:**
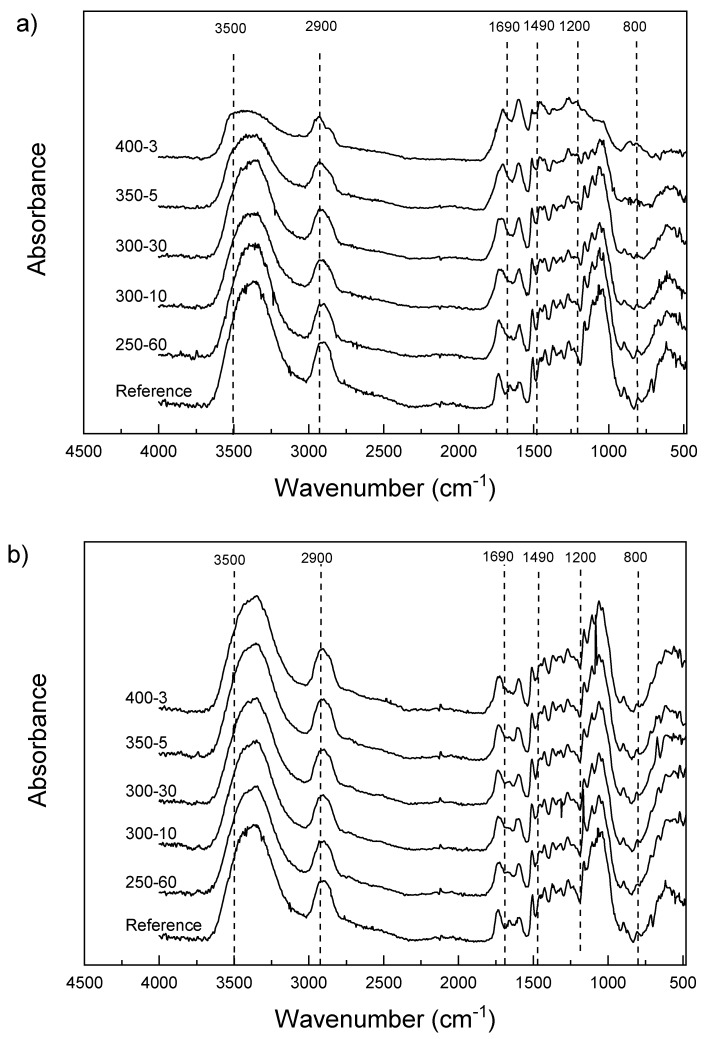
(**a**) FT-IR spectra measured from the surface and (**b**) the pyrolysis zone.

The PAS-FTIR spectra taken from the sample surfaces show changes characteristic for thermally modified wood (Figure 5a). The intensity of the OH stretches approximately 3500 cm^−1^ decreases notably with increasing temperature and the peak shifts to a higher wavelength. A shoulder appears in the bands representing the CH stretching of unsaturated and saturated hydrocarbons around 2900 cm^−1^ with increasing temperature. The area between 1690 and 800 cm^−1^ contains the major carbohydrate and lignin bands [19,33,34] that shows increased aromaticity. In the fingerprint area, there is a change in shape of the spectra at around 1490 cm^−1^, with distinct broadening with higher temperatures. The samples modified at 400 °C also show broadening at around 1210 and 1188 cm^−1^, which is characteristic of phenolic ring/phenolic OH group vibration [35]. The shapes of the bands in the carbohydrate area, including the wavelengths attributed to CO stretching (around 1040–1050 cm^−1^), CO stretching and OH in plane bending (1240–1250 cm^−1^), C-O-C and glucose ring stretching (1170 and 1130 cm^−1^) change especially with the 350-5 samples and disappear completely at 400 °C. The pyrolysis zone spectra show far less changes compared to reference sample (Figure 5b). The differences between modifications are almost unnoticeable, as was expected. The minor changes are mainly in the same areas as seen in Figure 5a (the OH and aromatic areas). The carbohydrate area seems practically unchanged. However, one interesting result can be seen in the behaviour of the sample 300-30. The averaged spectrum exhibits similar changes in the shape as the more severely modified samples in the surface spectrum, that is, slight broadening of the OH peak and the in the CH-stretching range as well as minute changes in the glucose ring structure at 1100 cm^−1^. It is evident that extended exposure time affects the depth and intensity of thermal degradation in the pyrolysis zone, which may be an indication of improved performance in terms of sorption. 

### 3.7. Cupping

**Figure 6:**
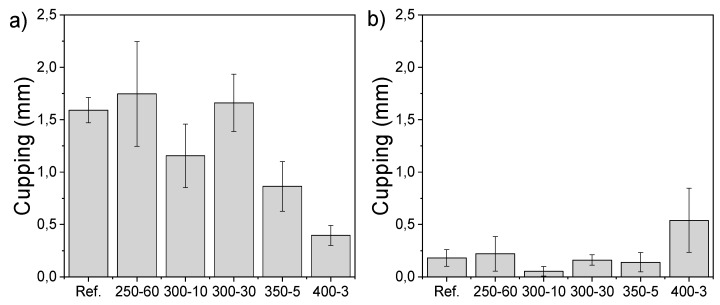
(**a**) Cupping (mm) after 72 h of water floating measured from the sap side, i.e., back of the sample and (**b**) the pith side, i.e., the modified surface of the samples.

The cupping measured on the sap side (back of the sample) of the laboratory samples after water floating shows reduced dimensional stability with longer holding times during charring (Figure 6a). On the other hand, the pith side (surface) of the modified sample cups more with the lowest holding time and highest temperature of 400-3 after 72 h of water floating (Figure 6b). It was shown that modification regime affects the cupping significantly (one way ANOVA, *p* = 0.000). The effect of varying sample thickness was taken into consideration and the Pearson correlation showed a dependence of −0.38 (*p* = 0.001) between thickness and extent of cupping. The multivariate generalised linear model calculated for both the back and surface of the samples after 72 h of water floating and corrected with sample thickness showed that modification regime had a significant effect on cupping of all samples (*p* < 0.05) and of all but reference and 400-3 (*p* < 0.005).

### 3.8. Density Profile

**Figure 7:**
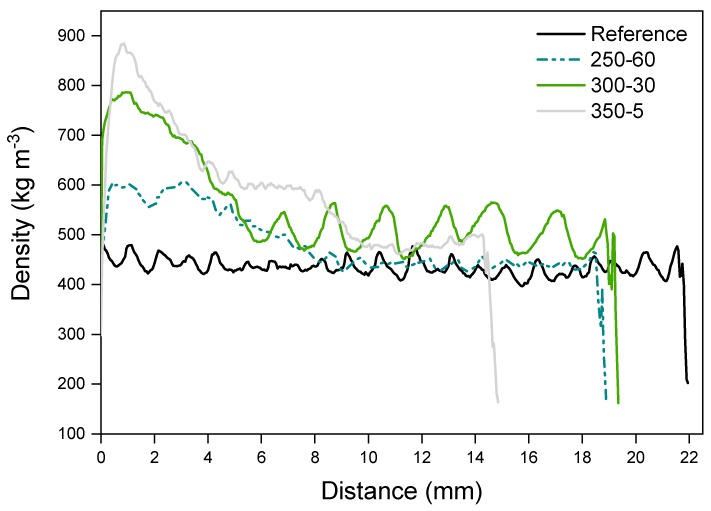
Density (kg m^−3^) of charred samples measured at the distance of 0–22 mm from the charred surface. The figure including standard deviations is presented in Supplementary Figure S2.

The density profile was measured from the reference, along with samples 250-60, 300-30 and 350-5 to study the combined effect of temperature and holding time on the wood. Density of wood correlates with dimensional stability, that was here measured by cupping after wetting. The pressure was held at the same level with all measured samples. The density peak forms on the surface layers of the modified samples but moves slightly inwards with the more severe treatments (Figure 7). 

## 4. Discussion

During the manufacturing of the samples, it was seen that some pressure is required to keep the wood samples flat against the hot surface. While moisture evaporates the annual rings of wood will seek to straighten, which leads to cupping on the sapwood side and the wood samples were curving away from the heated surface. The high surface pressure (1 MPa) set on the laboratory press was more than enough to fight the stresses, whereas a lower pressure may lead to an unevenly charred surface which has an effect on weathering. While the necessary pressure should be carefully examined, surface densification in general has been shown to be an effective way to increase the surface hardness and overall mechanical properties of wood [36,37]. Especially lighter wood species benefit from the process. As mentioned in the results, temperature profile may be slightly different in the compressed samples, as the surface density is higher. This may further hinder the conduction of heat into the sample and therefore effect the depth of the surface char layer and the pyrolysis layer.

The surface layer of charred wood acts as an efficient insulator that protects the interior by limiting the conduction of heat to the charring sample. The surface is, however, prone to cracking due to shrinkage and cupping and thus high tensions develop [20,27,38]. Sudden exposure to high temperature causes a rapid release of volatiles that may also tear the surface [27]. These cracks markedly increase the penetration of heat into the pyrolysis zone [25]. Crack development is dependent on the exposure time, as well as density of the sample [24]. Otherwise, density has not been unanimously proven to have an effect on the charring rate [24,25,26]. On the other hand, density (and density profile) of wood has a major effect on the other properties, namely sorption and dimensional stability. Wood densified from one surface has an asymmetric profile that is a likely contributor to cupping [39]. The procedure used here led to a sharp density peak at the surface of the sample that may create additional stresses with moisture exposure. The intensity of the peak was clearly more connected to the modification temperature instead of holding time, though in the SEM images it was seen that the compressive effect continued much further into the wood with 300-30 than the other samples. In wood densification, it has been noticed that the press closing speed affects the peak location and height. Slower closing speed allows the heated surface to dry, as well as for the heat and moisture to penetrate deeper into the sample with a softening effect [40]. Higher density correlates with higher surface hardness and elastic recovery that in this case is situated at the very surface of the compressed samples. The slight shifting of the peak of the sample modified at 350 °C indicates reduced surface hardness due to wood turning to a more porous, lighter char. The simultaneous compression left the surface intact but especially the samples modified at more severe conditions did crack, the 400-3 samples visibly after charring and the samples at and above 300-30 with exposure to moisture, some deeper and some more shallow. Changes related to dimensional stability during weathering cannot therefore be ruled out, though the cupping of some modified samples was reduced from reference. 

Along with density, the wetting rate of cell walls is an important parameter considering the time-dependent cupping behaviour of wood [41]. The cupping of wooden cladding boards has been reported to be an important factor limiting the service life of wooden facades [12] and reducing the dimensional instability is of high relevance when trying to reduce the life cycle costs of materials. Fast wetting of the surface in contrast to the interior creates tensions that either lead to checking or compressive strains. Charring reduced capillary absorption in comparison to reference samples. The dimensional stability measured by cupping following the water floating experiment was negatively affected by a longer holding time but higher temperature showed improved stability. Longer holding time in modification may cause higher stresses that are released in water sorption. Set-recovery of densified wood can be minimized by using saturated steam [36] and it is possible that post-conditioning of also charred wood could affect the extent of cupping. It is also worth noting that longer holding time leads to thicker pyrolysis zone beneath the charred surface, which behaves like TM wood with restricted sorption that in some conditions is also shown to cup less [12]. 

There are several changes taking place in the wood structure during thermal degradation that decrease the sorption capacity. One is the reduction in OH groups, seen also in the FT-IR spectra of the surface but also cross-linking in the carbohydrate-lignin matrix [21]. Wannapeera et al. [42] reported cross-linking in leucaena wood already below 275 °C (1 h, under nitrogen), that increased with longer holding times. The migration and enrichment of extractives to and on the surface also affect the surface wettability [36], though most fats and waxes disappear at temperatures above 180 °C [43]. The FT-IR bands around 2900 cm^−1^ show reduced intensity and the peak shifts slightly to the left indicating changes in the hydrocarbons originating from extractives but clear changes in the characteristic peak of fatty acid esters at 1740 cm^−1^ can be seen only above 300 °C, indicating there may still be some of these compounds left in the mildly treated samples. The restricted volatilization in compression may have some effect. The softening of lignin at elevated temperatures may also enable the diffusion of extractives deeper into the wood [43] that could leave some of these hydrophobic components present in the modified samples. The contact angle data support the speculation, as the samples modified at or below 300 °C show very consistent and stable behaviour, whereas the samples modified at 350–400 °C have increased wettability. Previously discussed cracking and increased porosity most likely have a major effect but chemical changes may also contribute.

Samples for SEM containing both hard intact wood and hard/brittle charcoal proved to be very difficult to cut without embedding. Adequate images were still obtained by soaking the samples in distilled water until saturation before cutting them with a microtome. This procedure reduced the amount of visible cell wall cracks noticeably. Without densification, the structure of the char is very similar to the structure of the wood. In fact, the structure remains so similar it is possible to identify the wood from which the charcoal is produced [20,38]. The ragged lumen edges reported in Kymäläinen et al. [30] were not seen in these micrographs—although the applied pressure crushed the cells close to the surface these structures were not seen in the uncompressed samples either. This could be related to different preparation methods (laser ablation vs. microtome). The changes in surface functional groups were consistent with previously reported results for wood. There is a reduction in the amount of hemicelluloses and glucose and an increase in aromatic compounds due the preferential loss of carbohydrates and increase of lignin. Heating to 250 °C showed only minor changes in the composition compared to reference, with the OH and CH peak intensities decreasing, as well as some small changes in the carbohydrate area, also reported by Rutherford et al. [34]. The infrared band at 3000 to 3600 cm^−l^ is the superposition of many hydroxyl groups absorbing at different states of hydrogen bonding [19]. With increasing temperatures, the intensity of these peaks decreases due to hydrogen bond OH stretching. The broadened peaks also shift slightly to the left. On the contrast, some shifting towards smaller wavelength can be seen with the peaks at 1740 cm^−1^ and below. The middle peak between 1740 and 1540 cm^−1^ of the reference sample disappears and the rest of the samples exhibit only two peaks. Chow & Pickles [19] accounted the shifting of peaks in this area to deacetylation and carboxylation at lower modification temperatures. At higher modification temperatures, this was speculated to be caused by formation of new carbonyls of carboxyls, or the destruction of crystalline lattice that reduces steric hindrance. Also, Rutherford et al. [34] accounted these bands to different carbonyl carbons. According to Labbe et al. [44] the region at around 1127–927 cm^−1^ is the most important in discriminating the different heat treatments. This area contains the absorption bands typically assigned to C–O stretching vibration in cellulose and hemicellulose, whereas lignin components are found at slightly higher wavelengths. Rutherford et al. [34] reported a distinct broadening of the lignin bands between 1600 and 1200 cm^−1^, seen also in this study, which probably represents skeletal vibrations of fused-ring aromatic structures. The disappearance of the peak at 1650 cm^−1^ of reference sample may coincide with absorption band of carbonyl or polyphenol and flavonoid compounds [19]. Comparison with literature would benefit from using powdered wood [32] but solid samples minimized dusting in the PAS-FTIR chamber. The band at 1600 cm^−1^ intensifies showing increased aromatic/double bonds. Aromatic condensation increases rapidly above 400 °C, where aromatic carbon reportedly accounts for over 90% of the charcoal carbon. Below 350 °C, the amount is less than half [45]. Increased aromatization reduces hydrophilicity but higher lignin content may affect the weatherability of the material negatively, as UV radiation is mainly absorbed by the lignin components. In the surface spectra, samples 300-10 and 300-30 are practically overlapping. However, the pyrolysis zone shows some minor changes in the otherwise linear composition of the spectra, with 300-30 exhibiting slightly reduced intensity in the OH and glucose ring stretching bands. This behaviour was also detectable in the DVS isotherms in Figure 4. A longer holding time stimulates progressive carbonization, increases the pH, decreases the negative surface charge [46] and seemingly intensifies the structural changes in thermal modification. 

Wood charcoal is a porous substance, a trait that has been successfully utilized in the application of activated carbons. However, the porosity develops differently in different wood species, atmospheres, temperatures and holding times. Heating wood under a nitrogen atmosphere, Rutherford et al. [34] showed that no porosity develops below 250 °C despite substantial loss of material through volatilization but the development coincided with the loss of aromatic carbon at high temperatures of over 350 or 300 °C after several hours. The development was speculated to take place only after conversion of aliphatic to aromatic carbon has ceased and aromatic carbon begins to degrade. Here, the charred surfaces created at above 350 °C were brittle to touch, which indicates porosity and therefore increased absorption. This proved to be an accurate assumption, supported also by the FT-IR spectra showing increased aromaticity. However, the increased wettability was evident only in the contact angle measurements, as the samples performed very well in water floating. The thermally modified layer was also thinner in the more severe modification regimes due to short holding time and the more severely charred samples showed fluctuation in the contact angles that follows from uneven sorption. It is possible that the overall hydrophobicity counteracts the porosity. Also, in the milder modification regimes adsorption to OH-groups forms a part of the overall mass gain by sorption, that may not show very clearly in the short-term (60 s of contact angle measurement) compared to the long-term (72 h or water floating). Overall, surface cracks affect thermal conductance in the charring phase but have a major effect also on the sorption behaviour of the wood. They may provide pathways into the unmodified wood that readily takes up moisture in the form of vapour and liquid. Swelling in the unmodified layers cause internal stresses that lead to dimensional changes and potentially to more cracks through checking and cupping. In some conditions, cracks also facilitate growth of fungal hyphae within the wood [47]. It was shown by Byrne & Nagle [19], that a slow heating rate reduces cracking. The heating rate was not controlled in this study but the surface was exposed instantaneously to the desired peak temperature. It is however feasible to assume that at lower temperature regimes less cracking occurs, although the dimensional stability measured by cupping was also increased at higher temperatures. Still, it might be of interest to investigate the possibility of a slower heating rate and its effect on the surface properties also at high temperatures. 

In actual charcoal manufacturing the charring of wood is seen to begin at 400 °C, as the rates of biomass carbonization below this point are very slow [38]. However, wood heated at air above 400 °C will easily combust. To avoid this and still use higher temperatures as well as longer modification durations, the manufacturing space should limit oxidation, which would significantly increase costs. In air, the pyrolysis vapours also escape the surface quite fast, which limits secondary reactions that increase the yield of char [18,23,38]. In large samples, however, different decomposition reactions overlap and secondary reactions involving moisture and volatiles become more important [27]. The relevance of this in a modification process and in the formation of a durable surface should be studied.

## 5. Conclusions

One-sided surface charring notably reduced the liquid water and water vapour sorption of spruce wood samples in comparison to unmodified reference samples. The reduction of OH groups and polar components, increase in aromatic structures, most likely accompanied by the densification created a surface with reduced hygroscopicity. The chemical changes intensified with higher temperatures. The dimensional stability measured by cupping was more dependent on the holding time and increased at high temperatures with short holding times. Cupping on both sides of the sample (as in 400-3) leads to a relatively stable behaviour during wetting. On the other hand, lower temperatures (below 350 °C) and longer holding times (30 to 60 min) promoted the favourable sorption characteristics measured by contact angle, whereas higher temperatures gave good results in other sorption tests. This may be partly caused by the unevenness of the surface that has an effect on variation between different measurement points. Finding the correct combination of temperature and time would increase both the hydrophobicity and the dimensional stability. The possibilities of tuning the heating rate to reduce cracking at higher temperatures, optimizing the surface pressure for an ideal compression ratio, as well as investigating the porosity and actual weatherability of the surface charred wood remain for further research.

## Figures and Tables

**Table 1:** Time-temperature regimes used in charring.

Time (min)	Temperature (°C)	Code
**60**	250	250-60
**10**	300	300-10
**30**	300	300-30
**5**	350	350-5
**3**	400	400-3

## Supplementary Materials

The following are available online at http://www.mdpi.com/1996-1944/11/11/2083/s1, Figure S1: Wettability measured by contact angle during a measurement period of 60 s; Figure S2: Density (kg m^−3^) of charred samples measured at the distance of 0–22 mm from the charred surface.
