# Supplementary material for: Sorption-Related Characteristics of Surface Charred Spruce Wood
Source: Materials (Basel). 2018 Oct 24;11(11):2083. doi: 10.3390/ma11112083 (PMC6266808; doi:10.3390/ma11112083)
Supplement: Supplementary file 1 [file materials-11-02083-s001.pdf]

Supplementary file 1.

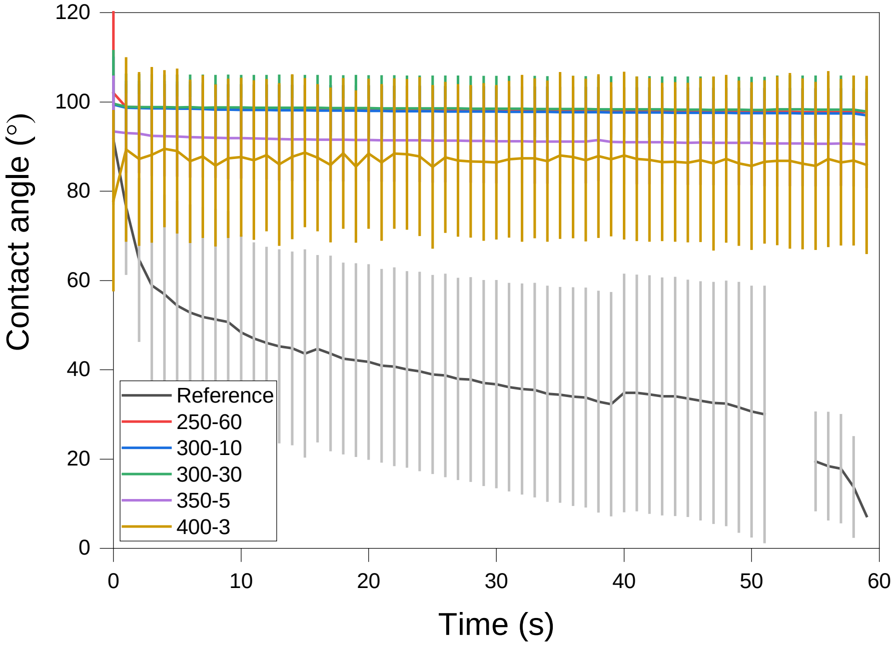

Figure S1. Wettability measured by contact angle during a measurement period of 60 seconds.

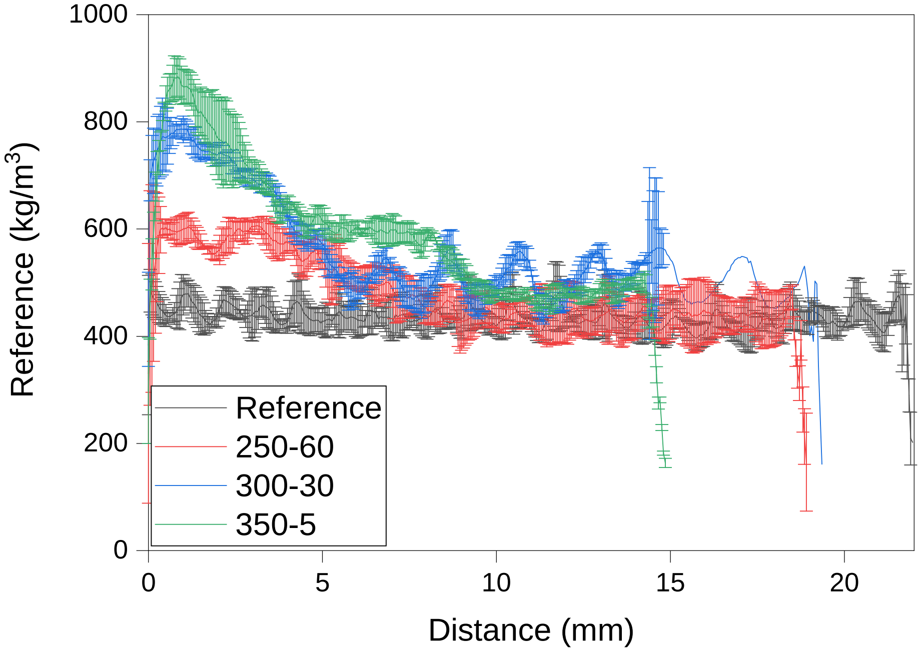

Figure S2. Density (kg m^{-3}) of charred samples measured at the distance of 0–22 mm from the charred surface.
